# Comparison of CpG Island Methylator Phenotype (CIMP) Frequency in Colon Cancer Using Different Probe- and Gene-Specific Scoring Alternatives on Recommended Multi-Gene Panels

**DOI:** 10.1371/journal.pone.0086657

**Published:** 2014-01-21

**Authors:** Marianne Berg, Hanne R. Hagland, Kjetil Søreide

**Affiliations:** 1 Department of Gastrointestinal Surgery, Stavanger University Hospital, Stavanger, Norway; 2 Centre of Organelle Research, University of Stavanger, Stavanger, Norway; 3 Department of Clinical Medicine, University of Bergen, Bergen, Norway; Howard University, United States of America

## Abstract

**Background:**

In colorectal cancer a distinct subgroup of tumours demonstrate the CpG island methylator phenotype (CIMP). However, a consensus of how to score CIMP is not reached, and variation in definition may influence the reported CIMP prevalence in tumours. Thus, we sought to compare currently suggested definitions and cut-offs for methylation markers and how they influence CIMP classification in colon cancer.

**Methods:**

Methylation-specific multiplex ligation-dependent probe amplification (MS-MLPA), with subsequent fragment analysis, was used to investigate methylation of tumour samples. In total, 31 CpG sites, located in 8 different genes (RUNX3, MLH1, NEUROG1, CDKN2A, IGF2, CRABP1, SOCS1 and CACNA1G) were investigated in 64 distinct colon cancers and 2 colon cancer cell lines. The Ogino gene panel includes all 8 genes, in addition to the Weisenberger panel of which only 5 of the 8 genes included were investigated. In total, 18 alternative combinations of scoring of CIMP positivity on probe-, gene-, and panel-level were analysed and compared.

**Results:**

For 47 samples (71%), the CIMP status was constant and independent of criteria used for scoring; 34 samples were constantly scored as CIMP negative, and 13 (20%) consistently scored as CIMP positive. Only four of 31 probes (13%) investigated showed no difference in the numbers of positive samples using the different cut-offs. Within the panels a trend was observed that increasing the gene-level stringency resulted in a larger difference in CIMP positive samples than increasing the probe-level stringency. A significant difference between positive samples using ‘the most stringent’ as compared to ‘the least stringent’ criteria (20% vs 46%, respectively; p<0.005) was demonstrated.

**Conclusions:**

A statistical significant variation in the frequency of CIMP depending on the cut-offs and genes included in a panel was found, with twice as many positives samples by least compared to most stringent definition used.

## Introduction

Cancer is a genetic disease caused by accumulation of molecular changes and modifications on DNA that drives tumorigenesis [1]. Proper molecular characterisation of the cancer geno- and phenotypes is important to reveal markers for early detection, prognostication or prediction, as well as increase the understanding of disease processes that may yield information for therapeutic intervention. Colon cancer is a well investigated cancer model, for which tumours are now separated into three phenotypical subgroups, depending on the predominant type of genetic aberrations: chromosomal instability (CIN) refers to changes at the chromosome level (e.g. copy number changes); microsatellite instability (MSI) refers to alterations in basepair-repeats (e.g. base change in the dinucleotide repeat CA_6_), and; CpG island methylator phenotype (CIMP) denotes an aberrant methylation spectrum (e.g. hypo- or hyper methylated cytosines in the promoter region), compared to normal cells [2], [3].

CpG-island methylation testing has been proposed as a tool for cancer detection, prognosis, and detection of residual disease in both blood or other body fluids [4], and indicates that methylation status is of clinical relevance [5]–[9]. Furthermore, methylation specific assays are commercially available today to detect colorectal cancer, either as testing for methylation of Vimentin in faeces samples or testing for methylation of *SEPT9* in blood [6], [10].

In recent years, attention has been focused on the biology and potential clinical importance of the CpG island methylator phenotype (CIMP) in CRC [11], [12]. While it is generally well accepted that etiologically and clinically distinct subgroups exist [13], [14], a precise definition of CIMP remains to be established, both methodologically and on a molecular level [15]. The increasing use of high-throughput technologies also for methylation studies have shown great impact and suggested several panels, *e.g.* to aid in the discrimination of colorectal tumour from epithelial cells, differentiate between disease stages, prognostic groups, and subgroups associated to other molecular features [16]–[18]. The lack of consensus for determining the CIMP phenotype is partly due to the fact that the cause of altered methylation pattern largely remains unknown [13], [19].

As a consequence, multiple gene panels, laboratory techniques, and marker threshold values are currently in use, all of which may result in differences in the reported prevalence of CIMP [20]. Since there is no universal standard or consensus on quantifying the phenotype, establishing its true prevalence is a challenge and hampers comparison between studies. Thus, the aims of this study was to systematically test several criteria for defining CIMP in colorectal cancer specimens, and investigate the difference in CIMP frequency when altering score levels.

## Methods

### Human Material and Study Ethics

Cancer tissue was obtained from colon cancer patients undergoing surgery at the Department of Gastrointestinal Surgery, Stavanger University Hospital, Norway. Patients were recruited to a prospective sentinel node trial, and all consented to study participation prior to inclusion and tissue retrieval. The Western Norway Regional Health Authority approved the use of human participants after having written informed consent, approval #197.04. All data were handled according to the Helsinki declaration.

Tissue was obtained as previously described [21], and fresh-frozen biopsies stored at -80 degree Celsius. A sample set of 64 stage II and III patients, and 2 colon cancer cell lines (Caco2 and HT29), were arbitrarily selected for evaluation in the current study.

### DNA Isolation

Fresh frozen cancer tissue samples, from an equal number of patients were included. Also, a reference sample of normal colonic epithelium was obtained ensuring a considerable distance outside the resection margin of the colon tumor. The DNA samples were extracted using DNeasy Mini kit or AllPrep DNA&RNA Mini Kit (Qiagen, Hilden, Germany).

### Methylation-specific Multiplex Ligation-dependent Probe Amplification (MS-MLPA) for CIMP Status

From all 64 DNA samples, 100 ng DNA were denatured in a total volume of 5 µl Tris-EDTA buffer, and further performed as recommended by the supplier (MRC-Holland, Amsterdam, the Netherlands).

Methylation-specific multiplex ligation-dependent probe amplification (MS-MLPA) is a method for simultaneous detection of methylation at several positions in one reaction [22], [23]. In short, a mixture of probe-mix (ME042-B1 CIMP, for more information on the specific probes see Table S1) and buffer were added to the denatured DNA, and probes were allowed to hybridize to the DNA at 60 C for 16 hours. Each sample was divided in two tubes, in which one half was ligated, and the other was ligated and digested using the methylation-sensitive restriction enzyme *HhaI*. Both samples were subsequently subjected to a PCR reaction using a thermal cycler (GeneAmp 2700, Applied Biosystems, Foster City, CA, USA), and fragment analysis performed on a capillary sequencer (ABI 3130*xl*, Applied Biosystems, Foster City, CA, USA). DNA from normal colonic mucosa was used as normal reference. The output from the analysis, after inter- and intra-sample normalization, is a percentage of methylation in the sample.

### Data Analysis of MLPA Data

The raw data from the fragment analysis were analysed using the Coffalyser.NET™ Software, beta version, (MRC-Holland, Amsterdam, the Netherlands). In short, the methylation of each position in every sample relative to the reference was calculated using Coffalyser.net™ software, with default settings.

All quality measures/parameters were within satisfactory range. All samples were normalised against multiple runs of the reference sample (inter sample normalisation), and furthermore, all probes were adjusted to reference probes within each sample (intra sample normalisation).

For methylation scoring of all probes within all samples, the ratio of peak height of digested versus undigested sample were calculated individually in Coffalyser, and the percentage of methylation used for further analysis.

#### Definitions and cut-offs

In order to investigate differences in CIMP frequency, which depended on the degree of methylation, different scoring parameters were tested.

Two panels of genes were investigated. The Ogino scoring panel includes 8 genes (*CACNA1G*, *IGF2*, *NEUROG1*, *RUNX3*, *SOCS1*, *CDKN2A*, *MLH1* and *CRABP1*), whereas the Weisenberger panel includes 5 of the above-mentioned genes (*CACNA1G*, *IGF2*, *NEUROG1*, *RUNX3* and *SOCS1*). For the Weisenberger panel (called Weisenberger hereafter) a positive score for three or more of the five genes is regarded CIMP positive, whereas for the Ogino panel two different definitions were tested; CIMP positivity if 5 of 8 genes were positive (called Ogino 5/8), and CIMP positivity if 6 of 8 genes were positive (Ogino 6/8).

Furthermore, different cut-offs for scoring positive methylation for each probe, as well as different definitions of the total methylation status for a specific gene were investigated: All positions/probes were scored using two different levels of methylation (≥20% or ≥30%) for defining a specific position/probe as methylated. Three different cut-offs were tested for defining methylation for each gene; either at least 33% (1/3) of probes were methylated; at least 66% (2/3) were methylated, or; having at least one or more methylated probes within the gene.

For each gene, several methylation sites were studied, ranging from 3 to 6 sites per gene. The number of methylation sites for the genes examined was as follows: 3 probes each for *RUNX3*, *CACNA1G* and *IGF2*; 4 probes each for *MLH1*, *CRABP1*, *SOCS1* and *CDKN1A*, and; 6 probes for *NEUROG1*.

**Figure 1:**
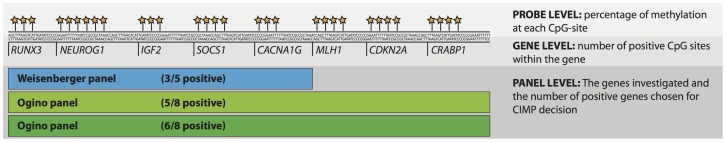
Illustration of the three levels of scoring, the panels and genes, and number of probes investigated in the presented study.

For CIMP decision, the three panels were used in combination with all the probe- and gene-wise cut-offs, Figure 1. This resulted in a total of 18 alternative scoring definitions for CIMP. Differences in positive samples were observed, and the statistical significance evaluated.

The ‘most stringent criteria’ for scoring of CIMP were defined as methylation of at least 30% on probe level, at least 66% positive probes within a gene, and 6 out of 8 positive genes in the Ogino panel. The ‘least stringent criteria’ were defined as 20% methylation on probe level, one or more positive probes within a gene, and 3 of 5 genes scored as positive in the Weisenberger panel.

#### Statistical analysis

All statistical analyses were performed by SPSS v21 (IBM Corp., Armonk, NY, US).

The number of positive samples resulting from each of the different scoring criteria was compared using McNemars test on a 2×2 table. All tests were two-sided, and statistical significance indicated for p-values <0.05.

## Results

### Probe-wise Cut-off

**Figure 2:**
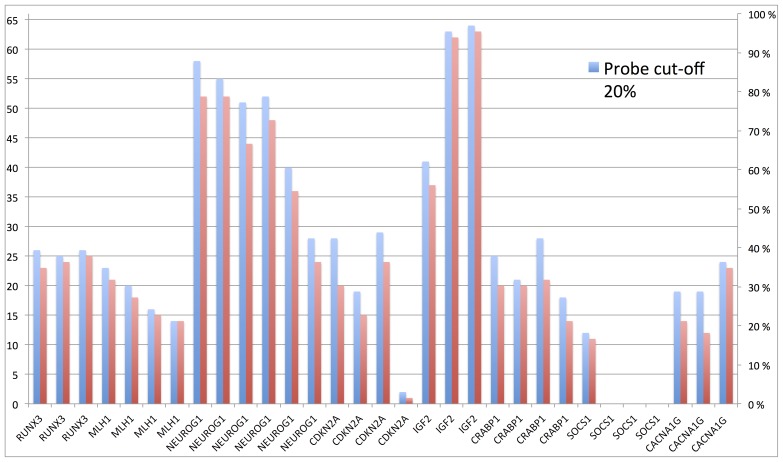
Scoring of methylated probes using a cutoff of 20% (red) and 30% (blue). The axis to the left shows the number of positive samples, and the axis on the right shows the percentage of positive samples.

For each probe, the relative methylation of the sample, as compared to a normal reference, was given as a percentage. The cut-off for scoring a probe as methylated was tested using both 20% and 30% methylation of sample vs reference, Figure 2. Both cut-offs were tested for all probes in the assay. Overall, using 30% as cut-off, the number of positive samples for the 31 different probes tested, varied from 0 (0%) to 63 (95%), whereas using 20% as cut-off, the variation was from 0 (0%) to 64 (97%). The mean variation in scoring of positive samples between 20% and 30% cut-offs were 3 (5%), varying from a minimum of 0 to a maximum of 8 samples (0–12%). Four of 31 probes (13%), in two different genes, showed no difference in the numbers of positive samples using the two cut-offs (03-037010228 in *MLH1*, 16-011256544, 16-011256960 and 16-011257200 in *SOCS1*). The largest variation was observed for probe 09-021965200 in *CDKN2A*, in which 8 patients (12%) were scored differently using the two different cut-offs. Three probes (16-011256544, 16-011256960 and 16-011257200 in *SOCS1*) did not have any positive samples using either of the criteria, as the methylation percentage varied from 0 to 5%.

### Gene-wise Cut-offs

The number of probes investigated for each gene varied from 3 to 6. Three different intra-gene settings were tested; one or more positive probes, more than 33% positive probes or more than 66% positive probes. All three settings were tested both for a probe-wise cut-off of 20% and 30%.

This resulted in six scorings per gene, for which a mean of 44.8% of the samples were scored as positive over the 8 genes. The mean percentage of positive samples across the genes, for each score criteria, ranged from 34.3% to 57.0%, using the most stringent (30% probe cut-off, 66% positive probes per gene), and least stringent criteria (20% probe cut-off, ≥1 positive probe per gene), respectively, Table 1.

**Table 1:** Methylation status for each gene based on different criteria.

	RUNX3	MLH1	NEUROG1	CDKN2A	IGF2	CRABP1	SOCS1	CACNA1G	Criteria Mean (%)
**P: 30% G: 66%**	24 (36)	14 (21)	42 (64)	5 (8)	61 (92)	19 (29)	0 (0)	16 (24)	34,3
**P: 30% G: 33%**	25 (38)	19 (29)	53 (80)	19 (29)	64 (97)	21 (32)	0 (0)	23 (35)	42,4
**P: 30% G: ≥1**	25 (38)	33 (50)	57 (86)	35 (53)	64 (97)	23 (35)	11 (17)	23 (35)	51,3
**P: 20% G: 66%**	26 (39)	15 (23)	48 (73)	7 (11)	63 (95)	20 (30)	0 (0)	22 (33)	38,1
**P: 20% G: 33%**	27 (41)	20 (30)	57 (86)	25 (38)	64 (97)	23 (35)	0 (0)	26 (39)	45,8
**P: 20% G: ≥1**	27 (41)	35 (53)	60 (91)	44 (67)	64 (97)	33 (50)	12 (18)	26 (39)	57,0
**Overall Mean (%)**	38,9	34,3	80,1	34,1	96,0	35,1	5,8	34,3	

Number, and percentages (in parentheses), of positive samples for each gene using different scoring criteria. The criteria mean column indicates the mean percentage of positive samples using the specific criteria, while the overall mean row indicates the percentage of positive samples per gene. P =  probe-level cut-off, G =  Gene-level cut-off.

**Figure 3:**
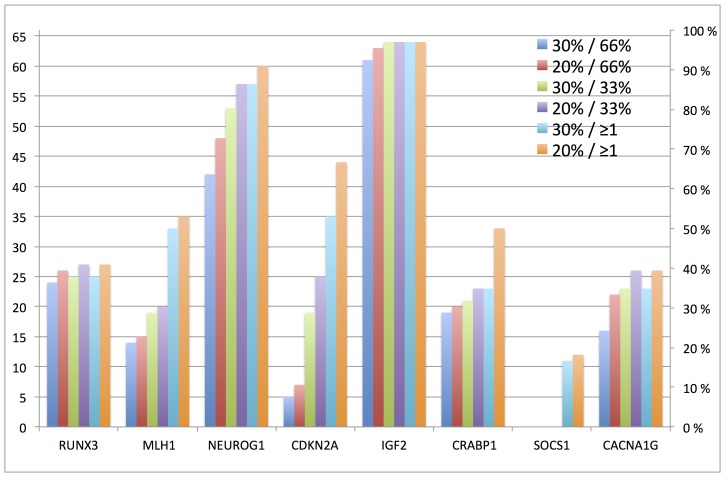
The number (left vertical axis) and percentage (right vertical axis) of samples scored as positive for each gene using all combinations of probe- and gene-wise cut-offs.

SOCS1 had the lowest number of positive samples, with a mean score of positive samples of 5.8%, ranging from 0 to 18.2%, whereas the mean score of positive samples for IGF2 was 96.0% for the 6 alternative criteria, ranging from 92.4 to 97.0%, Table 1/Figure 3.

*CDKN2A* showed largest variation in positive samples using the 6 alternative criteria. The number of positive samples varied by 39 samples, ranging from 5 to 44, using the most stringent and least stringent criteria, respectively. On the contrary, *RUNX3* and *IGF2* showed the least variation; in both cases only 3 patients were scored differently over the 6 different criteria. For *RUNX3* 24 patients were scored as positive using the most stringent criteria, compared to 27 patients with the least stringent criteria. For *IGF2* the number of positive samples was 61 and 64, using the most and least stringent criteria, respectively.

### CIMP Frequencies

**Figure 4:**
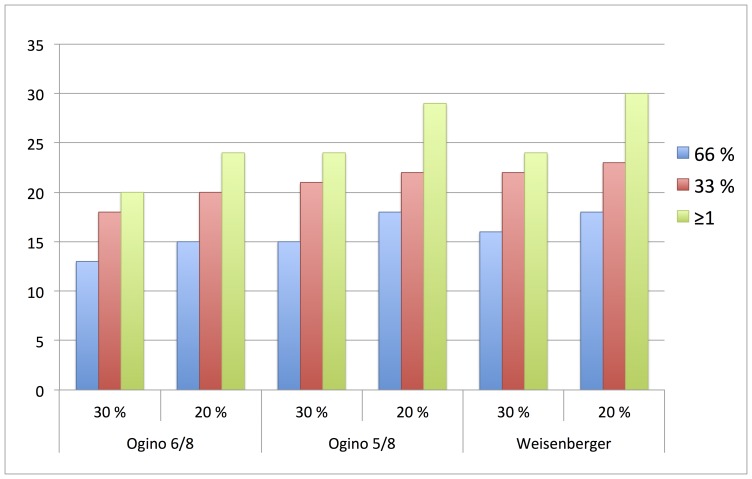
Number of positive patient samples using comparing alternative scoring probe-wise, gene-wise and panel-wise.

The outcome, in terms of number of positive samples using each of the three panels, including the different combinations of probe-wise and gene-wise scorings, are presented in Table 2/Figure 4.

**Table 2:** Array of CIMP positive samples using different alternative criteria for scoring of CIMP.

	Ogino 6/8		Ogino 5/8		Weisenberger	
	30%	20%	30%	20%	30%	20%
**66%**	13	15	15	18	16	18
**33%**	18	20	21	22	22	23
≥**1**	20	24	24	29	24	30

The number of CIMP positive samples combining the alternative scoring criteria on probe level (horizontal, 2^nd^ row), gene level (vertical left row), and using the alternative gene panels (horizontal 1^st^ row).

The most stringent criteria, using a probe cut-off of 30%, and demanding that 2 out of three (>66%) of the probes within a certain gene must be positive, in addition to using the Ogino 6/8 panel, returned 13 (20%) CIMP-positive samples. On the contrary, using a probe cut-off of 20%, requiring at least one or more positive probe within a gene, and the Weisenberger panel (3 out of 5 positive genes), returned 30 (45%) CIMP-positive samples.

When using the least stringent criteria on probe- (20%) and gene- (≥1 positive genes) level, 24 samples (36%) were scored as positive using Ogino 6/8-panel, 29 positive (44%) using Ogino 5/8-panel and 30 positive (46%) using Weisenberger panel. These probe- and gene level criteria resulted in the largest variation between the panels, resulting in 6 patients (9.1%) being scored differently. For 47 patient samples (71%), the CIMP status was constant and independent of criteria used for scoring; 34 samples were constantly scored as CIMP negative, and 13 scored as CIMP positive.

A significant difference between positive samples using the most stringent (20%) as compared to the least stringent (46%) criteria was demonstrated, p<0.005. When comparing the least stringent criteria on both probe-level (20%) and gene-level (≥1 positive probe) between the three panels, only the Ogino 6/8 compared to the Weisenberger panel showed a significant difference (p = 0.031). On the contrary, the most stringent criteria on probe- and gene level did not return significant differences between the panels.

Testing for differences within each of the panels, showed that increasing the stringency on gene-level from the least stringent (≥1) to the most stringent (66%), keeping the probe level constant, resulted in significant different number of positive samples. On the other hand, keeping the gene level constant, while changing the probe level, did result in significant different positive samples for the ≥1 criteria, within the Weisenberger panel only.

## Discussion

Independent of methodology used, criteria for scoring of CIMP would need to be defined at three levels; which genes should be tested, how many methylation sites should be affected for the gene to be transcriptionally inactivated, and to which extent a methylation site must be methylated to affect the transcription status of the gene.

This study found a significant difference in the CIMP frequency in colon cancer using MS-MLPA methodology, when comparing different criteria used for defining methylated samples. Although a high percentage of samples (71%) were consistently scored (of which 13 were CIMP positive) with no change according to criteria used, the remainder were influenced by a change in scoring criteria set. Notably, only four of 31 probes (13%) investigated showed no difference in the numbers of positive samples using the different cut-offs. The largest variation was observed for one of the probes in the *CDKN2A* gene. For the 8 genes investigated, *RUNX3* and *IGF2* were most consistent with little variation among samples, while considerable variation was noted for *CDKN2A*, in accordance to results reported by Ogino et al. [20]. The criteria used resulted in significantly different number of positive samples when used for the two suggested panels to define CIMP status in the literature, namely the Ogino- and the Weisenberger panels [20], [24].

The findings pose several important implications. For one, it demonstrates variation between the frequency of CIMP depending on the cut-offs and genes investigated in a panel. This should be noted when investigators use CIMP as part of the classification suggested for colorectal cancer. Second, it demonstrates variability in methylation within and across genes. This may either be related to true differences within genes and the investigated probes, or related to technique used for identification. If the former is true, the reason to why certain sites are (more consistently) methylated while others are not should be investigated, as it may form new knowledge about carcinogenesis and how methylation may contribute to cancer development. Further, it may be that genes and probes (such as *RUNX3* and *IGF2*) should be used together with other or similarly consistently methylated genes in panels to form decision on CIMP status, in order to avoid over- or underestimation of CIMP positive or negative samples.

The presented study was conducted in order to evaluate the relevance of using alternative scoring criteria for CIMP, by using methylation specific multiplex ligation-dependent probe amplification (MS-MLPA). In the published literature there are several studies evaluating gene panels and CpG positions to be used for CIMP scoring, but the underlying cut-offs for the specific position and gene are poorly discussed [11], [20], [24].

In this study, the difference in number of positive samples using the most stringent as compared to the least stringent criteria was statistically significant, and indicates that the chosen cut-offs used for scoring of CIMP status is important. Furthermore, statistical analyses also showed that combinations of all three levels of scoring alternatives gave statistically different number of positive samples, implying that all three levels should be carefully evaluated. However, within all three panels, changing the gene level criteria had a larger impact on the number of samples scored as positive than changing the probe level criteria. Even if several different methods could be used to detect the CIMP phenotype, all these methods will have to consider the scoring criteria underlying the CIMP definition. Independent of methodology used, one will have to consider which CpG sites to study and define a cut-off for methylation of the specific CpG site, define how many CpG sites within the gene which will need to be methylated to define the gene as methylated, and finally, which genes, and how many of them will need to be methylated to eventually score the sample as methylated.

Differences in CIMP scoring between gene panels have been evaluated [20], and found to be quite small (<3.2%), even between panels using different genes. Also, a comparison of cut-off for the Ogino panel (5 of 8 and 6 of 8) was compared, and 3% of the samples showed different scoring (18% and 15%, respectively) [20].

In general, independent of criteria and method used for scoring of CIMP, the degree of methylation measured for a given position would depend on the homogeneity of the cells, and infiltration of normal cells in the sample studied.

Also, the choice of normal reference sample for each study should be carefully evaluated, as the methylation of a sample is relative to the chosen reference. In this study we used a sample from normal epithelium from a colon cancer patient. In theory, this sample could harbour some epigenetic features influencing the results of the methylation pattern in the cancer samples. However, this will affect all samples equally, and is therefore of minimal relevance with regards to evaluating scoring criteria. Given the opportunity, the best option would have been to test normal mucosa tissue from the patient where the tumour tested originates from alongside the tumour itself. Given the limited material we had available, our best option was to use the before-mentioned control and thus present the data accordingly.

Most reports of CIMP status have used bisulphite treatment of the DNA prior to the chosen method of interrogation of the methylation pattern. This manipulation of the DNA converts unmethylated cytosines to uracils, while the methylated are left unconverted. There are several protocols for bisulphite treatment, differing in the bisulphite concentration used and incubation/conversion time, and add another level of complexity to the conclusive CIMP status. In the study presented herein, no DNA treatment has been done prior to the MLPA method, thus limiting the source of errors.

Further, the fact that methylation of gene are fluctuating naturally in order to regulate gene expression, and that the nature of the epigenome, contrary to the genome, is to be in constant transformation, is another aspect complicating a clear definition of CIMP [25]. To circumvent this challenge one would have to rely on material that have been collected at the same time interval and stage in the patients treatment, as such avoiding possible time-lapse discrepancies.

## Conclusions

In conclusion, this study illuminates how different scoring criteria result in statistically significant different number of CIMP positive samples. The definition of CIMP in colorectal cancer is not yet agreed upon, neither on a qualitative level (which CpG islands/genes that should be tested), on a quantitative level (how many positions needs to be methylated), nor on a technical level (what methodology is to be used). However, irrespective of the methodology used one would have to decide on cut-offs for positive scoring, which should be carefully evaluated, as the resulting CIMP frequency will be significantly influenced by this decision. Further research into this important area is warranted to improve clinical translation of the role of CIMP in cancer.

## Supporting Information

Table S1 Sequences, chromosomal location, and fragment lengths for the investigated positions/probes in the MLPA method.
(DOCX)
