# Supplementary material for: Comparison of CpG Island Methylator Phenotype (CIMP) Frequency in Colon Cancer Using Different Probe- and Gene-Specific Scoring Alternatives on Recommended Multi-Gene Panels
Source: PLoS One. 2014 Jan 21;9(1):e86657. doi: 10.1371/journal.pone.0086657 (PMC3897740; doi:10.1371/journal.pone.0086657)
Supplement: Table S1 — Sequences, chromosomal location, and fragment lengths for the investigated positions/probes in the MLPA method. (DOCX) [file pone.0086657.s001.docx]

| **Gene name** | **Frag. length** | **Chr. location** | **Mapview^§^** | **5' probe sequence** | **3' probe sequence** |
| --- | --- | --- | --- | --- | --- |
| RUNX3 | 346 | 01p36.11 | 01-025.128720 | CCGGTGGACGTGCTGGCGGACCACGCA | GGCGAGCTCGTGCGCACCGACAGCCCCAACTTCCTCT |
| RUNX3 | 372 | 01p36.11 | 01-025.128920 | CCGCTTGGGTCTACGGGAATACGCAT | AACAGCGGCCGTCAGGGCGCCGGGCAGGCGGA |
| RUNX3 | 256 | 01p36.11 | 01-025.129597 | GCTAGAAATTTGCTTAGAACGTCCGGGTC | CCACGGAAGGCGCCCTTGCCGCCCTCTCT |
| MLH1 | 355 | 03p22.2 | 03-037.009360 | TCCGCCACATACCGCTCGTAGTAT | TCGTGCTCAGCCTCGTAGTGGCGCCTGACGTCGCGTT |
| MLH1 | 463 | 03p22.2 | 03-037.009621 | CTGCTGAGGTGATCTGGCGCAGA | GCGGAGGAGGTGCTTGGCGCTTCTCAGGCTCCTCCTCT |
| MLH1 | 132 | 03p22.2 | 03-037.009760 | CAAGAGCGGACAGCGATCTCTAACGCGCAA | GCGCATATCCTTCTAGGTAGCGGGCAGTAGCCGCTTCAGG |
| MLH1 | 177 | 03p22.2 | 03-037.010228 | GACACGCCTCTTTGCCCGGGCAGA | GGCATGTACAGCGCATGCCCACAACGGCGGAGGC |
| NEUROG1 | 166 | 05q31.1 | 05-134.898938 | TGCGTCCAGGGCCGCGTTCAA | GTTGTGCATGCGGTTGCGCTCGCGATCGTTGGCCTTG |
| NEUROG1 | 282 | 05q31.1 | 05-134.899244 | GTGTCCGTCGGTCCTGCACAGCGCAAC | GATGCCAGCCCGCCTTGAGACCTGCATCTCCGACCTC |
| NEUROG1 | 211 | 05q31.1 | 05-134.899351 | GGCCGCCAGGGCGCACTTACGT | TCCCAACAGCCTGGGGTTGTTACTCTGTGCCAGTTGCGGG |
| NEUROG1 | 389 | 05q31.1 | 05-134.899479 | CTGATCTGATCGCCGGCGACATCA | CTCAGGAGACCGGCCGGGCGCGTGGCCC |
| NEUROG1 | 364 | 05q31.1 | 05-134.899537 | CCCATTGTTGCGCCGGGTACTTA | AGGGGTCCTGAGGCCAGTCGTGTGCCACACTCGGTGCT |
| NEUROG1 | 202 | 05q31.1 | 05-134.899663 | CCTCATCCCCGTGCAGCGCCCGGGTATTTGCATAAT | TTATGCTCGCGGGAGGCCGCCATCGCCCCTC |
| BRAF | 409 | 07q34 | 07-140.099560 | CCTTTACTTACTACACCTCAGATATATTTCTTCATGAAG | GAAATCTCGATGGAGTGGGTCCCATCAGTTTGAACAGTTGTCTGG |
| CDKN2A | 232 | 09p21.3 | 09-021.964677 | CACCTGGATCGGCCTCCGACCGTAAC | TATTCGGTGCGTTGGGCAGCGCCCCCGCCTCCAGCAGC |
| CDKN2A | 183 | 09p21.3 | 09-021.965200 | CTTTTAACAGAGTGAACGCACTCAAACACGCCTTTGCT | GGCAGGCGGGGGAGCGCGGCTGGGAGCAGGGAGGC |
| CDKN2A | 335 | 09p21.3 | 09-021.984268 | GCAGGTTCTTGGTGACCCTCCGGA | TTCGGCGCGCGTGCGGCCCGCCGCGAGTGAG |
| CDKN2A | 195 | 09p21.3 | 09-021.985276 | GGAAGAGGAAAGAGGAAGAAGCGCTCAGAT | GCTCCGCGGCTGTCGTGAAGGTTAAAACCGAAAATAAAAATGG |
| IGF2 | 171 | 11p15.5 | 11-002.117594 | TCAAGCCACCTGCATCTGCACTCA | GACGGGGCGCACCCGCAGTGCAGCCTCC |
| IGF2 | 418 | 11p15.5 | 11-002.118681 | CCACCGCCTGCCACAGAGCGTTCGATCGC | TCGCTGCCTGAGCTCCTGGTGCGCCCGCGGAC |
| IGF2 | 141 | 11p15.5 | 11-002.118895 | GAAATTTCTCTCTAGCGTTGCCCAAACACA | CTTGGGTCGGCCGCGCGCCCTCAGGACGTGG |
| CRABP1 | 207 | 15q25.1 | 15-076.419820 | GCCACCATGCCCAACTTCGCCGGCAC | CTGGAAGATGCGCAGCAGCGAGAATTTCGACGAGCTGC |
| CRABP1 | 310 | 15q25.1 | 15-076.420033 | GCTGAACGCGTGGGTTCCGGGATCTCT | ACCAGCTTCTCCGAGACCCGGTGCGCCTGGGAGACAA |
| CRABP1 | 265 | 15q25.1 | 15-076.420493 | GTGGAGATCCGCCAGGACGGGGATCAG | TTCTACATCAAGACATCCACCACGGTGCGCACCACTG |
| CRABP1 | 319 | 15q25.1 | 15-076.420701 | CCTTTGCAGCCTGTGGCGCGCCTTCCT | TGCAGGGTGTGTACACTGGCTGTTTGCAGAGGGGGTTTGTGCATCCTAG |
| SOCS1 | 239 | 16p13.13 | 16-011.256544 | CCGATTCTACTGGGGGCCCCTGAGCGTGCACG | GGGCGCACGAGCGGCTGCGCGCCGAGCCCGT |
| SOCS1 | 154 | 16p13.13 | 16-011.256960 | GACTTGGTGCTCCGTGCTCGCCCCCT | AGGGCCGGGTCCGCCGGGAGCGCCGCCCT |
| SOCS1 | 399 | 16p13.13 | 16-011.257200 | CCTTTCTCCGGCCCTAGCCCAAATCGCCCA | GACCAGGCGCGGATCCCAGCCTGGCCAGCAGGCGGCG |
| SOCS1 | 300 | 16p13.13 | 16-011.257552 | CCAGCCCCGCCTCCGAGCCGGTTTAAA | AGACTGGCGCAGGGGCGGGCGCCGAACAGAGCGA |
| CACNA1G | 273 | 17q21.33 | 17-045.993509 | GAGCCTGGGCGCGAAGCGAAGAA | GCCGGAACAAAGTGAGGGGGAGCCGGCCGGC |
| CACNA1G | 246 | 17q21.33 | 17-045.993744 | CGGGCGATCCGGAGAGGGGCA | AGCGGCGCCCCTCAGAGGAGGTGTCCTCACGCAA |
| CACNA1G | 218 | 17q21.33 | 17-045.993972 | GCGGCTGTCCTGGCTCAAGTAGAAGAA | AACCACCGGGGCCAGCGCCGGGTACGGC |

^§^Refers to hg18
